# Effect of Gas Composition on Surfactant Injectivity in a Surfactant-Alternating-Gas Foam Process

**DOI:** 10.3390/molecules29010100

**Published:** 2023-12-22

**Authors:** Jiakun Gong, Yuan Wang, Raj Deo Tewari, Ridhwan-Zhafri B. Kamarul Bahrim, William Rossen

**Affiliations:** 1 College of Mechanics and Materials, Hohai University, Nanjing 210098, China; 2 Department of Geoscience and Engineering, Delft University of Technology, 2628 CN Delft, The Netherlands; w.r.rossen@tudelft.nl; 3 College of Water Conservancy & Hydropower Engineering, Hohai University, Nanjing 210098, China; wangyuan@hhu.edu.cn; 4 PETRONAS, Kuala Lumpur 50088, Malaysia; raj.tewari@petronas.com (R.D.T.); ridhwan_zhafri@petronas.com (R.-Z.B.K.B.)

**Keywords:** foam, surfactant-alternating-gas, injectivity, gas composition, solubility

## Abstract

Aqueous foam is a dispersion of gas in liquid, where the liquid acts as the continuous phase and the gas is separated by thin liquid films stabilized by a surfactant. Foam injection is a widely used technique in various applications, including CO_2_ sequestration, enhanced oil recovery, soil remediation, etc. Surfactant-alternating-gas (SAG) is a preferred approach for foam injection, and injectivity plays a vital role in determining the efficiency of the SAG process. Different gases can be applied depending on the process requirements and availability. However, the underlying mechanisms by which gas composition impacts injectivity are not yet fully understood. In this work, the effect of gas composition on fluid behavior and injectivity in a SAG process was investigated using three gases: N_2_, CO_2_, and Kr. Our observations revealed that gas solubility in liquid was key for the formation and evolution of liquid fingers, and therefore was very important for liquid injectivity. A lower gas solubility in liquid led to a slower increase in surfactant solution injectivity. In addition, the development of surfactant solution injectivity took significantly longer when the surfactant solution was partially pre-saturated compared to when it was unsaturated. Additionally, the propagation of the collapsed-foam bank during gas injection was accelerated when the gas had a greater solubility in water.

## 1. Introduction

Aqueous foam is a dispersion of gas in liquid, where the liquid acts as the continuous phase and the gas is separated into bubbles by thin liquid films called lamellae [1,2]. Foam presents unique rheological properties as it flows through porous media, in particular reduced gas mobility. For this reason, in a variety of applications, foam is injected into geological formations, such as aquifer (or soil) remediation [3,4], CO_2_ sequestration [5,6], and enhanced oil recovery [7,8].

Foam is primarily introduced into geological formations through two methods: the co-injection of a surfactant solution and gas [9] and the injection of alternating slugs of gas and a surfactant solution, a technique known as SAG (surfactant-alternating-gas) [10]. The SAG method is usually preferred due to its operational benefits, which include lower pipe and facility corrosion, improved operational safety, and superior gas injectivity [11]. The success of the SAG process relies heavily on the injectivity of a surfactant solution, which can pose challenges. The high injection pressure during liquid injection can cause the injection well to fracture [12,13], and reducing the injection rate to avoid fracturing slows the process, increasing costs and reducing benefits [14]. In a CO_2_ sequestration process, a reduced injection rate can reduce the foam sweep efficiency and thereby reduce gas trapping by foam in the formation [15]. Therefore, ensuring injectivity, particularly for surfactant solution injection, is essential. Reservoir simulation models for foam processes do not represent foam injectivity accurately. Gong et al. [16] showed that they can miscalculate the near-well rise in the injection-well pressure by more than tenfold.

**Figure 1:**
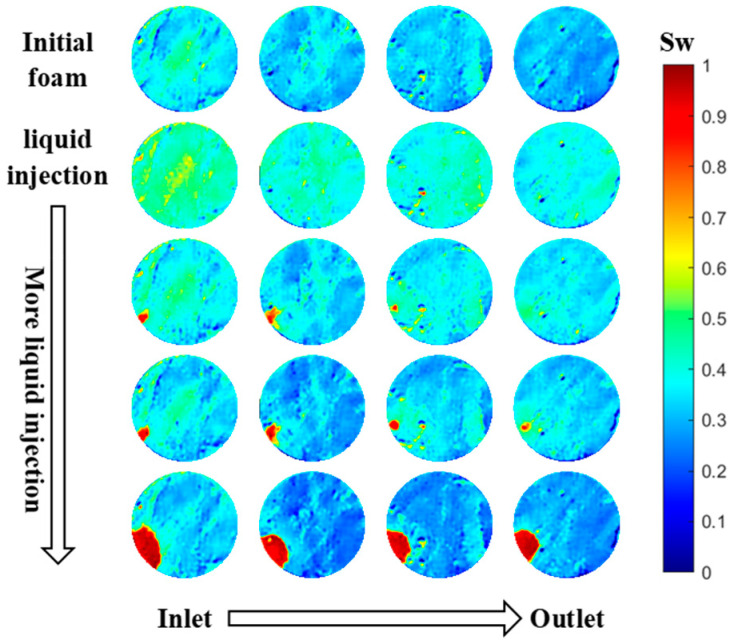
Cross-section CT images of aqueous phase saturation S_w_ at four locations within a core during the injection of a surfactant solution, subsequent to the establishment of steady-state N_2_ foam, based on the experiments in earlier works [17]. The liquid saturation within the liquid fingers approached 100% due to the dissolution of trapped gas within the fingers.

The injectivities of gas and surfactant solutions during SAG operations are dominated by the intricate flow dynamics occurring in the vicinity of the well [16,17]. This includes the flow of the gas phase, liquid phase, and foam bubbles, as well as the interactions between each. Coreflood experiments [17] showed that the surfactant solution first entered a core full of foam with a low relative permeability and a high injection pressure. The pressure gradient then experienced a modest increase before remaining stable for a period of time. Subsequently, it fell abruptly behind a front that propagated from the entrance to the exit of the core. This behavior was visualized using X-ray computed tomography. As demonstrated in the CT scan images shown in Figure 1, when the surfactant solution was injected into foam, it penetrated the foam in fingers, pushing out some gas inside the fingers, while trapping gas outside the fingers in place. Over time, the gas trapped within the liquid fingers dissolved into the unsaturated injected surfactant solution, greatly increasing liquid mobility. The advance of the fingers of nearly a 100% liquid saturation fit the advance of the zone of greatly increased mobility seen in the pressure measurements. In addition, a material balance of the gas dissolved into unsaturated liquid fit the rate of advance of the fingers. Over time, as the liquid fingers dissolved the trapped gas surrounding the fingers, they gradually expanded outward. Gas dissolution, leading to a huge increase in liquid mobility, strongly affects the evolution of surfactant solution injectivity [17].

During the injection of the gas slug in the SAG foam process, a bank of dried-out, collapsed-foam forms in the vicinity of the well and propagates very slowly downstream. The rate of advance of the collapsed-foam region of increased mobility fits the expected rate of evaporation of water into the gas [16,17]. The surfactant solution injected afterwards first fills the region where the foam has collapsed, with relatively high mobility. It then proceeds to penetrate the trapped-foam region downstream through the fingers described above [17]. Thus, the gas properties play a key role in the development of gas and surfactant solution injectivity in the SAG foam processes.

Different types of gas can be utilized in foam applications depending on the process objectives and availability in the local area. These include steam, CO_2_, CH_4_, and N_2_ [18,19,20]. Previous studies have revealed that the dynamics of foam behavior are strongly impacted by gas composition. The stability of an individual foam lamella is influenced by gas composition, as varying gases exhibit distinct intermolecular interactions.

The impact of gas composition on the characteristics of steady-state foam has been extensively studied. However, to our knowledge, there is still a lack of understanding regarding the impact of gas composition on the injectivities of gas and surfactant solutions during SAG operations. Therefore, the objective of this study was to investigate the controlling mechanisms and the impact of gas composition on the injectivities of gas and surfactant solutions in the SAG foam process. The organization of this work is as follows. The results of the various injection scenarios are presented in Section 2, along with explanations for the observations. The materials and methods used in the experiments are outlined in Section 3. Section 4 summarizes the conclusions derived from this work.

## 2. Results and Discussion

### 2.1. Influence of Gas Composition on Steady-State Foam Strength

**Figure 2:**
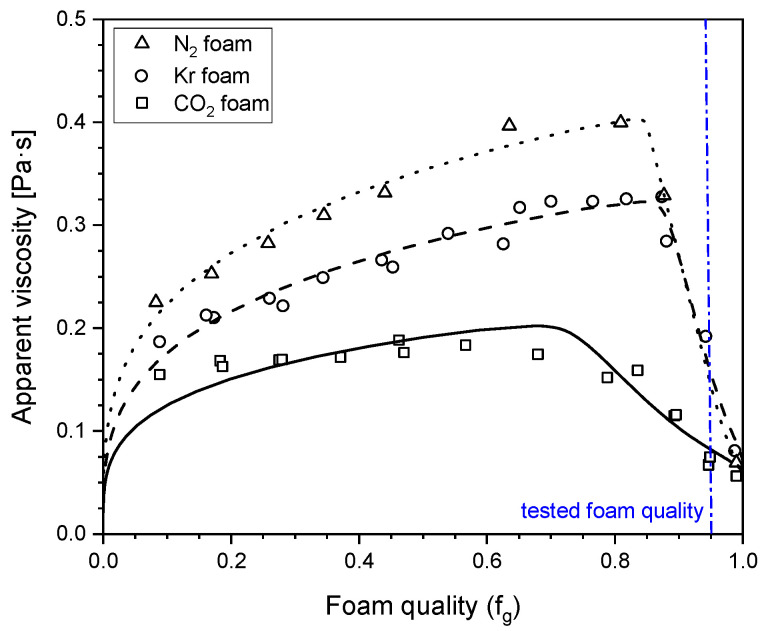
The dependence of the apparent viscosity of foam at a steady state on the gas type and foam quality (injected volume fraction of gas).

The present research aimed to evaluate the stability of steady-state foam by quantifying its apparent viscosity. The foam apparent viscosity is the viscosity of a fluid that would give the measured pressure gradient in single-phase flow at the given injection rate. The foam apparent viscosity (*k*∇p/*u*_t_) was determined from the pressure gradient (∇p), the total superficial velocity (*u*_t_), and the permeability (*k*) by applying Darcy’s Law. The determination was based on a series of foam quality scan experiments (measuring the apparent viscosity as a function of the foam quality), as illustrated in Figure 2. The foam quality is the volume fraction of flowing gas in the injected fluids under the given experimental conditions. This plot enabled comparison of the foam mobility for various types of gas at varying gas fractions.

The models for the foam behavior for a given gas, surfactant solution, and experimental conditions as a function of the foam quality are described in the literature [21,22,23]. The behavior fell into two regimes: the “low-quality regime” at qualities below the point of maximum apparent viscosity, and the “high-quality regime” above that point. Briefly, the models assumed that the bubble size controlled the foam mobility. In the low-quality regime, the bubble size was fixed at roughly pore size. The behavior was shear thinning because of the shear-thinning rheology of moving bubble trains and bubble mobilization with an increasing pressure gradient. As the gas fraction increased, the foam dried out until it reached the “limiting capillary pressure” [17]. Beyond this point, the foam collapsed progressively as the gas fraction increased.

As shown in Figure 2, for each type of gas, the steady-state foam presented both flow regimes. Figure 2 shows that, for a given foam quality, the greatest foam strength at a steady state belonged to N_2_ foam, while Kr foam and CO_2_ foam ranked second and third in order. The strengths of N_2_ foam and Kr foam were similar in the high-quality regime.

### 2.2. Impact of the Gas Composition on the Surfactant Solution Injectivity Directly Following Foam

**Figure 3:**
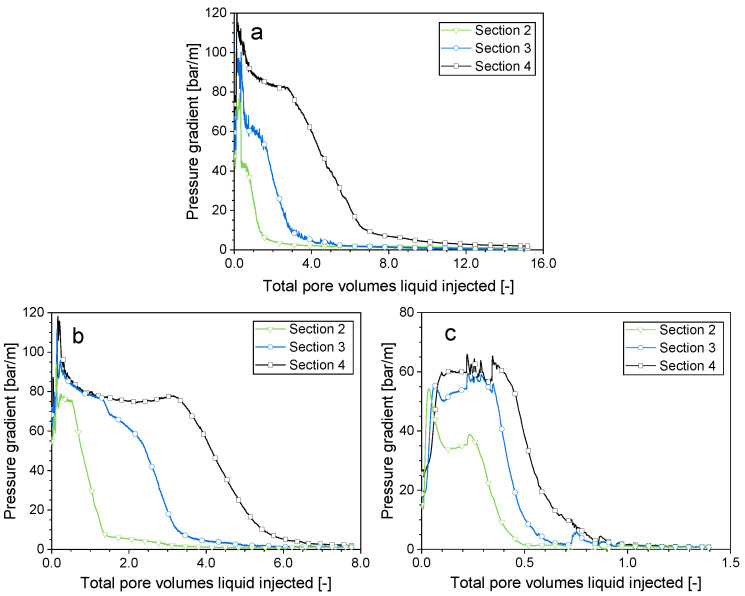
Sectional pressure gradients during surfactant solution injection following steady-state foam (*f*_g_ = 0.95). (**a**) Nitrogen foam, (**b**) krypton foam, (**c**) carbon dioxide foam.

In this section, the investigation focused on exploring the influence of the gas composition on the flow behavior during the injection of a surfactant solution subsequent to achieving steady-state foam. The initial steady-state foam quality was 0.95 for each of the cases examined. Since injection of a surfactant solution directly followed the foam injection, this could be considered the worst case for surfactant solution injectivity when the foam is placed into a formation in a SAG manner. As presented in Figure 3, the pressure gradient changed in a similar pattern with the various gases. The pressure gradient first increased sharply and was constant for a time, and then declined in a front that propagated from the entrance towards the exit of the core. As the front passed out of each section into the next, the pressure gradient in that section fell.

Since the pressure gradient changed in a similar pattern as more surfactant solution was injected with each gas, it was reasonable to conclude that, in general, the flow characteristics when injecting the surfactant solution after Kr foam and CO_2_ foam was similar to those of N_2_ foam, which were confirmed with CT imaging [17], as illustrated in Figure 1. The pressure gradient underwent three stages of development.

Initially, the pressure gradient was low, which indicated that the liquid penetrated the core with considerable ease. The pressure gradient then abruptly climbed to a maximum value. Since considerable gas was trapped, the liquid encountered great flow resistance.

Following that, the pressure gradient either remained constant for a time or decreased until it reached a plateau. Throughout this time frame, the liquid swept the entire cross-section and made its way to the outlet. In our experiments, the liquid broke through after a similar amount of surfactant solution injection for all the cases: 0.17 pore volumes (PVs) of injected liquid, which roughly corresponded to the point that the pressure gradient reached its peak value. The rapid breakthrough of the liquid reflected the gas trapped in the core.

The injection of the surfactant solution into N_2_ foam and Kr foam exhibited certain distinctions compared to the injection of the surfactant solution into CO_2_ foam. In the cases of N_2_ foam and Kr foam, the pressure gradient spiked to a peak before gradually decreasing to a steady state. In the case of CO_2_ foam, the pressure gradient increased directly to a plateau. The difference in the initial foam strength could be the reason. As shown in Figure 2, at the foam quality (0.95) in this study, the apparent viscosities of N_2_ foam and Kr foam were close to each other and were greater than that of CO_2_ foam. CO_2_ foam could be more mobile during the surfactant solution injection, which could mean that less gas was trapped when the liquid advanced to the outlet. Once the liquid broke through, it flowed through the core without displacing more foam bubbles. The pressure gradient was nearly constant. In contrast, in the cases of N_2_ foam and Kr foam, the liquid still displaced movable foam bubbles after breakthrough, which reduced the liquid flow resistance. Consequently, the pressure gradient declined moderately.

**Figure 4:**
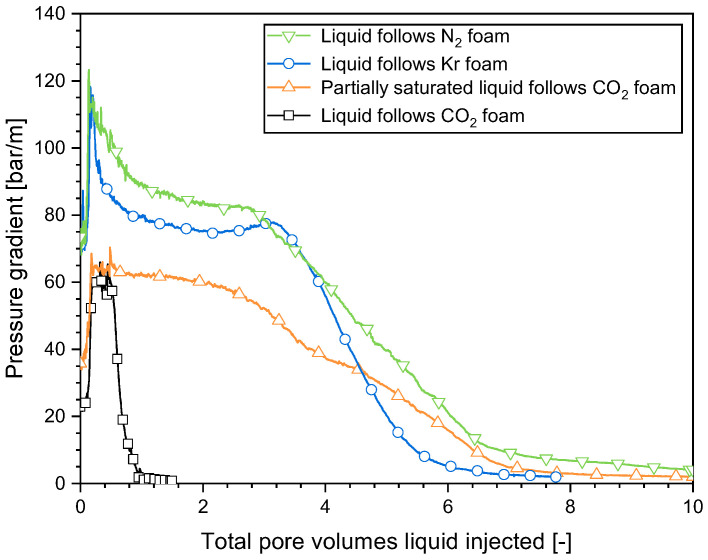
Pressure gradients in Sec. 4 during the injection of the surfactant solution following the establishment of steady-state foam (*f*_g_ = 0.95) with various types of gas.

As illustrated in Figure 4, the injection of an unsaturated surfactant solution following N_2_ foam had the greatest plateau value of the pressure gradient (approximately 85 bar/m), followed by Kr foam (approximately 75 bar/m). CO_2_ foam had the lowest plateau pressure gradient (approximately 65 bar/m). This order was in line with the apparent viscosity of the initial 0.95-quality foam (Figure 2). The unsaturated surfactant solution following CO_2_ foam had a plateau pressure gradient similar to that of the partially CO_2_-saturated surfactant solution following CO_2_ foam. Thus, the plateau value for the pressure gradient observed when injecting the surfactant solution after foam was evidently primarily influenced by the initial foam properties. The liquid was less able to mobilize the foam bubbles in a stronger foam or to find a way through the midst of the foam bubbles, resulting in a higher pressure gradient.

In the third stage, as more surfactant solution was injected, a front with a decreasing pressure gradient advanced from the entry toward the exit. As depicted in the CT scan images (Figure 1) of a case of surfactant solution injection following N_2_ foam [17], the liquid fingers formed, propagated, and expanded outwards as additional surfactant solution was introduced, while the gas outside of the liquid finger remained trapped. In fact, gas saturation rose around the fingers as the pressure there fells from the reduction in the pressure gradient. The water content within the liquid finger was nearly 100%, which greatly increased the water relative permeability. This, in turn, drove down the pressure gradient. Our earlier work [17] illustrated that the amount of N_2_ dissolved into the injected volume of the surfactant solution roughly accounted for the reduction in gas saturation inside the liquid finger. This indicated that gas dissolution is essential to the formation and development of the liquid finger.

The pressure gradient of Kr foam and CO_2_ foam evolved in a similar manner as that of N_2_ foam (Figure 3), which suggested that liquid fingering and gas dissolution happened in a similar way with those gases. However, the decline in the pressure gradient occurred at different rates for the various gases (Figure 4). The pressure gradient dropped rapidly to a small value within CO_2_ foam, while the pressure gradient within N_2_ foam declined most slowly. The propagation of the front could be described by a dimensionless propagation velocity, as proposed in our earlier work [16]. The dimensionless velocity is the fraction of the pore space behind the front divided by the pore volumes injected. It was 0.13 for N_2_ foam, 0.16 for Kr foam, and 0.87 for CO_2_ foam. The disparity can be explained by the varying solubility of the gases in water. An approximation of the N_2_ and Kr solubilities in water at the experimental temperature and pressure can be obtained from Henry’s Law [24]. The solubility of all three gases in brine differed from their solubilities in pure water. The CO_2_ solubility presented in Table 1 is the solubility in brine obtained from the experiments at similar experimental conditions [25]. At the experimental condition, CO_2_ had the greatest solubility, followed by Kr, while N_2_ had the lowest solubility (Table 1). The propagation velocity of the front of the pressure gradient decrease for the gases followed the order of their solubilities.

To further confirm the influence of the capacity of the liquid to dissolve gas on the evolution of surfactant solution injectivity, in one experiment the surfactant solution was partially saturated by pre-dissolving an amount of CO_2_. As illustrated in Figure 4, the reduction in the capacity of this liquid to dissolve CO_2_ significantly impacted the evolution of the pressure gradient. It dropped much slower in the case of the partially saturated surfactant solution following CO_2_ foam (after about a 2.4 PV injection) than that of the unsaturated surfactant solution injection following CO_2_ foam (after about a 0.3 PV injection, about seven times faster).

In summary, surfactant solution injectivity directly following foam was significantly influenced by the gas composition and, in particular, the solubility of the gas in the liquid. During the injection of the surfactant solution after foam, the evolution of the pressure gradient occurred at a faster rate with an increasing solubility of the gas in the liquid.

### 2.3. Impact of the Gas Composition on the Foam Collapse during Gas Injection

**Figure 5:**
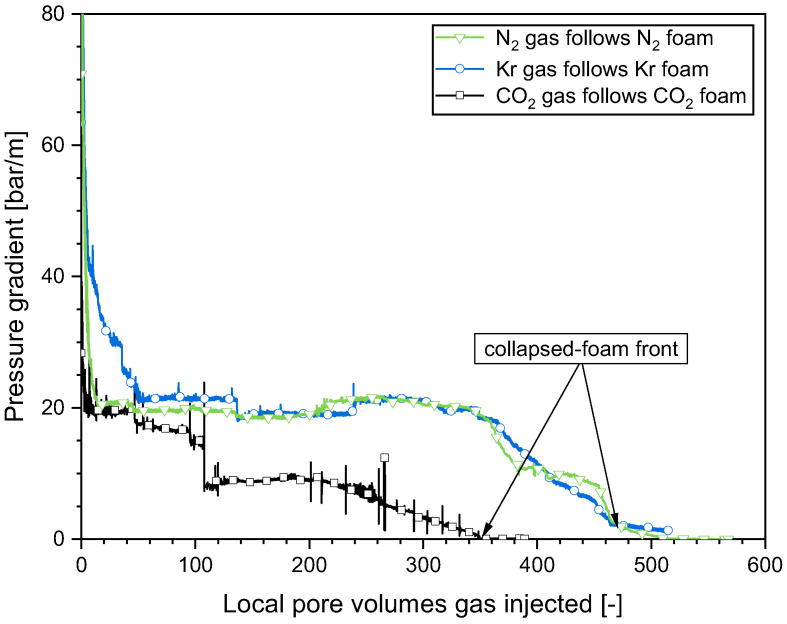
The spread of the collapsed-foam front with various types of gas. To illustrate the dimensionless propagation velocity of the collapsed-foam front, the pressure gradients are presented in relation to the local pore volume (LPV), i.e., the injected gas volume divided by the cumulative pore volume from the injection point to a given position [16]. The dimensionless velocity at which the front of a bank propagates can be expressed as 1/(LPV).

It was revealed in our previous study that the foam weakened or collapsed in the near-well region when subjected to prolonged gas injection during the SAG operation [17]. In the present section, how the gas composition affected the bursting of foam bubbles during the gas injection was explored. Three types of gas, N_2_, Kr, and CO_2_, were injected following N_2_ foam, Kr foam, and CO_2_ foam, respectively. As presented in Figure 5, the pressure gradients in all the cases examined evolved in a similar manner. Initially, the pressure gradient first declined to a plateau, which implied foam weakening, then remained remarkably stable over an extended time. The plateau pressure gradients for the three cases were comparable, which indicated that the weakened foams were similar to each other during this period. The pressure gradient then dropped further as additional gas was introduced, indicating the drying out and bursting of foam bubbles after an extended period of gas injection. This most likely implied a shift towards “continuous-gas foam” as opposed to the complete elimination of soap films [26]. In the CT experiments with N_2_ foam, the gas saturation in the “collapsed-foam” zone was approximately 0.9 [17], though the liquid mobility was much greater than with full-strength foam. The collapse of the foam as it dried out was consistent with the “limiting capillary pressure” model for foam stability [21]. With the injection of more gas, the collapsed-foam front advanced gradually but steadily from the entrance to the exit of the core. For N_2_ foam and Kr foam, the leading edge of this front advanced with a comparable dimensionless velocity, approximately 1/520. By comparison, the collapsed-foam front advanced somewhat quicker in the case of CO_2_ foam, with a dimensionless velocity of approx. 1/350. N_2_ and Kr could be considered nearly ideal gases. Water evaporates into an ideal gas to equalize its vapor pressure. A total of 1 PV of dry N_2_ or Kr evaporated approx. 4.18 × 10^−4^ PVs of liquid water under our experimental conditions. According to the published experimental data and a newly developed equation of state [27], the water solubility in CO_2_ under our experimental condition was about 1.5 times of that in N_2_ and Kr, and approx. 6.27 × 10^−4^ PVs of water dissolved into 1 PV of CO_2_. This was consistent with the variation in the dimensionless propagation velocities of the leading edge of the collapsed-foam front for the cases with CO_2_ and N_2_ or Kr, i.e., about 1.5 times faster with CO_2_. We concluded that water evaporation into dry gas is a key factor in foam weakening or collapse, regardless of the type of gas. In addition, a pressure-gradient-driven flow might also play a role in foam drying and collapse. The strong effect of liquid solubility in gas implied that the injection pressure would decline more quickly during the gas injection with a CO_2_ foam.

### 2.4. Impact of the Gas Composition on Surfactant Solution Injectivity in a SAG Foam Process

**Figure 6:**
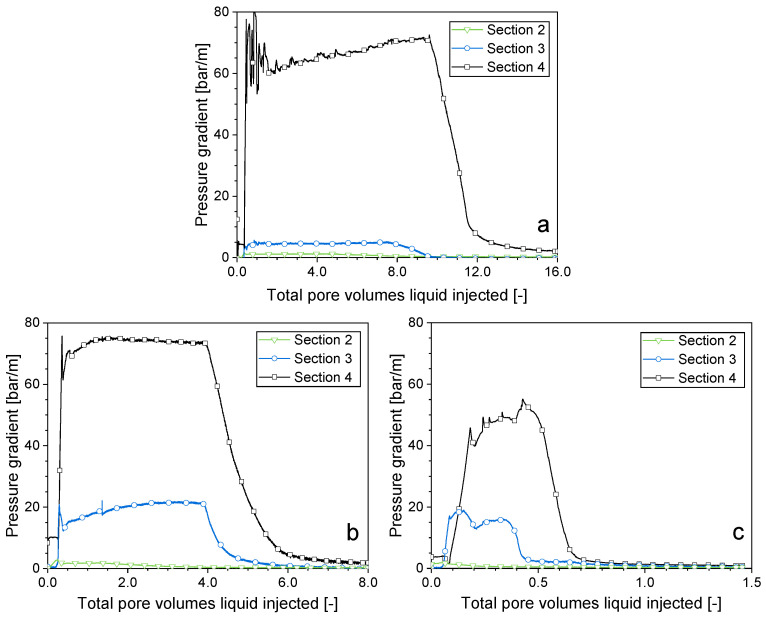
Injection of the surfactant solution following a similar amount of gas for various types of foam. (**a**) Nitrogen foam, (**b**) krypton foam, (**c**) carbon dioxide foam.

In the present section, how the composition of gas impacts the injectivity of surfactant solution subsequent to a time of gas injection was explored. Gas was injected following the establishment of steady-state foam, until the collapsed-foam front entered Sec. 3 of the core. This enabled the study of the pressure gradient during the flow of the surfactant solution into both the collapsed-foam region and the area beyond it with intact foam. As illustrated in Figure 6c, the surfactant solution injection following a time of CO_2_ injection resulted in a pressure gradient of less than 2 bar/m in Sec. 2, where the foam collapsed, while the pressure gradient approached 55 bar/m when the surfactant solution was introduced subsequent to steady-state CO_2_ foam (Figure 3c). The collapse of the foam during the gas injection greatly reduced the flow resistance during subsequent surfactant solution injection. With CO_2_ foam, the foam collapse reached part of Sec. 3. The pressure gradient was approx. 15 bar/m compared to approx. 60 bar/m when the liquid encountered full-strength foam (Figure 6c). In contrast, the peak pressure gradient in Sec. 4 beyond the region of foam collapse was hardly impacted by the preceding time of the gas injection and was analogous to that of the surfactant solution injection into full-strength foam. The cases with N_2_ foam (Figure 6a) and Kr foam (Figure 6b) showed phenomena similar to the case with CO_2_ foam. Therefore, regardless of the gas composition, a period of gas injection can weaken or even collapse the foam, greatly increasing the mobility of the following surfactant solution slug. In a field application, the region of collapsed foam would propagate slowly from the injection well, thereby enhancing the surfactant solution injectivity [16].

**Figure 7:**
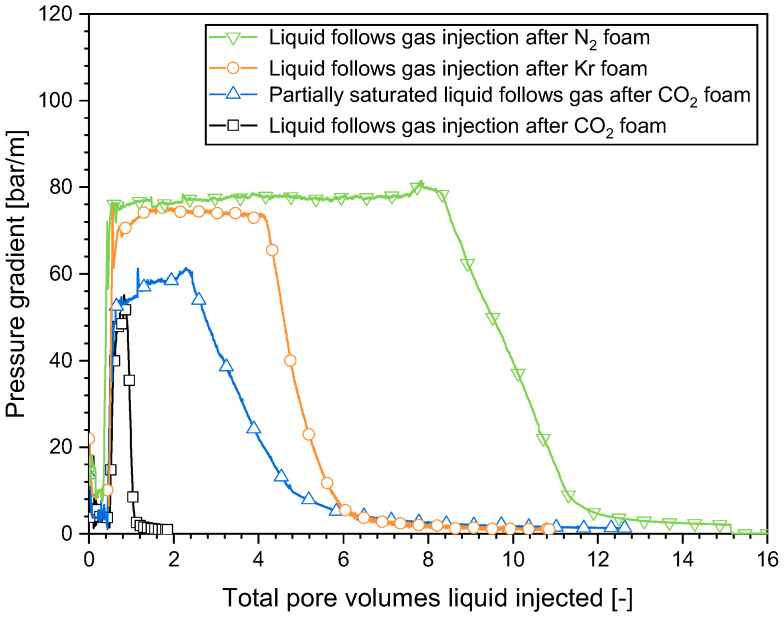
Pressure gradients in Sec. 4 when introducing the surfactant solution after a comparable volume of gas injection subsequent to different foam varieties.

The flow characteristics were significantly influenced by the type of gas when the surfactant solution was introduced into the area beyond the collapsed-foam region. This was similar to the effect observed when the surfactant solution was injected immediately after foam. As shown in Figure 7, for all the cases examined, the surfactant solution flowed in with a low mobility, reflecting the foam trapped in place. The pressure gradient then rose sharply until it reached a plateau and declined afterward as the gas dissolution front propagated downstream. The pressure gradient declined the quickest in the case of CO_2_ foam, followed by Kr foam, then N_2_ foam. This order corresponded to the gas solubility in water. For the partially CO_2_-saturated surfactant solution injected after a period of CO_2_ injection, compared to the injection of the unsaturated surfactant solution, the plateau in the pressure gradient lasted longer and the decline in the pressure gradient happened more slowly due to the reduced capacity for gas dissolution into the liquid.

When the surfactant solution injection followed the gas injection instead of following steady-state foam, Sec. 4 experienced a longer-lasting plateau in the pressure gradient and a more rapid subsequent decline. For example, in the cases of N_2_ foam, the plateau lasted for approx. 2.5 PVs, and the decline took about another 4 PVs when the surfactant solution was injected following steady-state foam (Figure 4). The plateau lasted for approx. 7.5 PVs and the decline took about 3 PVs when the surfactant solution was injected following a gas injection period (Figure 7). For CO_2_ foam, the plateau lasted for approx. 0.35 PVs, and the decline took about 0.45 PVs when the surfactant solution was injected following steady-state foam. The plateau lasted for approx. 0.4 PVs, and the decline took about 0.2 PVs when the surfactant solution was injected after a period of gas injection. The extended plateau in the pressure gradient could be ascribed to the gas accumulation within the collapsed-foam zone adjacent to the inlet, where the liquid penetrated and traversed the entire cross-section [17]. As noted above, the “collapsed-foam zone” had a higher gas saturation than full-strength foam, of order 0.9. There was more gas there to saturate the injected liquid; therefore, it took longer for the unsaturated liquid to reach the foam region beyond. The reason for the faster decline of the pressure gradient was unclear. It is possible that the liquid finger within the foam area during the surfactant solution injection following the gas injection might have been wider compared to that of the surfactant solution injection directly after steady-state foam, which led to a quicker dissolution of the gas into the liquid, as illustrated by the CT scan results in our previous work [17]. This is one possible reason why the decline in the pressure gradient happened more quickly when the liquid flows in after a gas injection period.

## 3. Materials and Methods

### 3.1. Experimental Materials and Apparatus

#### 3.1.1. Materials

The cylindrical Berea sandstone core samples used in these experiments were drilled from the same block. The cores had a diameter of 3.8 cm with a length 17 cm. Their porosity averaged approximately 0.21, with permeabilities of about 160 mD (1.6 × 10^−13^ m^2^). A surfactant solution (0.5 wt% concentration) was prepared by combining an alpha olefin sulfonate (AOS_C14–16_) surfactant and synthetic brine (3 wt% salinity), which included salts of potassium chloride, calcium chloride, sodium sulphate, magnesium chloride, and sodium chloride. To explore how the gas composition impacted the flow characteristics near the well during a SAG operation, three gases were employed: nitrogen, krypton, and carbon dioxide.

#### 3.1.2. Apparatus

**Figure 8:**
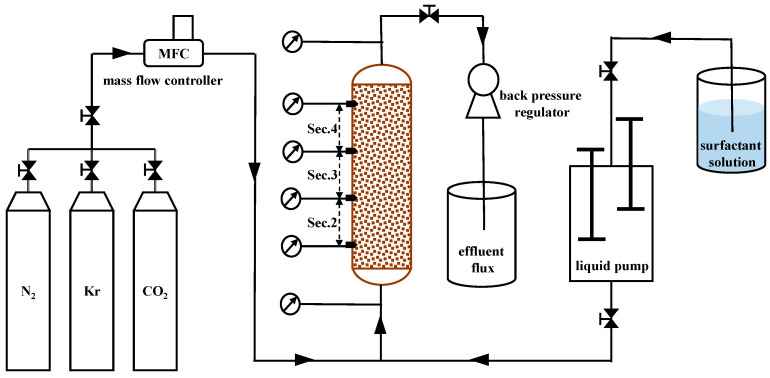
Schematic representation of the experimental apparatus.

Figure 8 depicts the experimental setup. To prevent flow bypassing, the core was coated with epoxy and housed in a core holder made of polyether ether ketone (PEEK), which was vertically positioned in an oven to ensure that the experimental temperature remained at approximately 90 °C. Nitrogen, krypton, or carbon dioxide gas was introduced into the core from the bottom via a mass flow controller, while a Quizix pump controlled the injection rate of the surfactant solution, also from the bottom of the core. To sustain a back pressure of 40 bar, a back-pressure regulator was employed. The injection rates and foam quality (volume fraction of gas in the injected fluid) were fixed for each measurement, with the gas volume calculated at the back pressure. To monitor the absolute pressures and pressure drops across the core sections, a total of six pressure transducers were utilized. In this work, our attention was directed towards Sec. 2–4, each of which measured 4.2 cm in length, in order to exclude the entrance region and the capillary end effect near the core outlet face [28].

### 3.2. Experimental Method

At the beginning of each experiment, a vacuum was imposed on the core. Multiple pore volumes (PVs) of CO_2_ were injected initially, followed by another application of a vacuum. Afterward, degassed water was introduced into the core. The back pressure was then raised to the experimental pressure (40 bar). A total of 10 PVs of brine was injected to displace and dissolve any remaining CO_2_, followed by a 5 PV surfactant solution to ensure the complete saturation of the core and ensure that no further adsorption of the surfactant on the core would occur during the experiment. The core was then ready for the experiments.

Three groups of dynamic experiments were conducted as follows.

(1) Influence of the gas composition on the steady-state foam strength. To conduct this analysis, the steady-state pressure gradient was measured with the co-injection of gas and a surfactant solution at a series of foam qualities (volume fraction of gas in the injected fluids) and a fixed injection rate: a “foam quality scan” (Figure 2), i.e., a measurement of steady-state foam mobility in the core as a function of the foam quality. By computing the injection rate and foam quality, the gas volume was based on the gas density at the core back pressure. The dissolution of CO_2_ in the surfactant solution was factored into the CO_2_ foam quality calculation, given the high solubility of CO_2_ in water. These tests were conducted with a total superficial, or “Darcy”, velocity of 2 ft/day (equivalent to 7.1 × 10^−6^ m/s). The foam mobility in the core was quantified as the “apparent viscosity”, i.e., the viscosity of a fluid that would give the same pressure gradient at the given injection rate. In these experiments, steady-state foam was established within the first section of the core. This was clear in the pressure gradient measurements, which were similar in Sec. 2–4 and much greater than in Sec. 1. The models for the foam behavior for a given gas, surfactant solution, and experimental conditions as a function of foam quality were described in our previous work [21,22,23].

(2) Impact of the gas composition on the injectivity of the surfactant solution immediately after foam. Following the establishment of steady-state foam at a quality of 0.95, the surfactant solution was injected. The cases investigated included the injection of an unsaturated surfactant solution following the establishment of N_2_, Kr, or CO_2_ foam, and one case with a surfactant solution 60% saturated with CO_2_ injected following CO_2_ foam. Previous CT studies [17] established the link between the measured pressure gradient and the mechanisms described in Section 1. During the gas injection, the collapse of the foam occurred behind a slowly advancing front. During the liquid injection, the liquid first invaded the collapsed-foam region and then formed and advanced as liquid fingers with a nearly 100% liquid saturation. Therefore, we used the pressure gradient measurements in the sections along the length of the core to infer the advance of the various fronts across the core. The dimensionless velocity of the front here meant the pore volume of the core up to the position of the front, divided by the pore volumes of the fluids injected up to that time.

(3) Impact of the gas composition on the injectivity of gas and a surfactant solution in a SAG process. After achieving steady-state foam in the core by the co-injection of gas and a surfactant solution, a large slug of gas was injected followed by a large slug of a surfactant solution. The purpose was, first, to see the effect of prolonged gas injection on foam stability, specifically the growth of the collapsed-foam bank from the core inlet, as described above. Then, during the surfactant solution injection, we observed the effect of the prolonged surfactant solution injection following gas. This step revealed the liquid advancing through the collapsed-foam bank, then penetrating the intact foam bank downstream, and finally the formation and advancement of liquid fingers with a very high liquid mobility. The dimensionless velocities of the various fronts were again quantified using the dimensionless velocity. N_2_, Kr, and CO_2_ were used for the gas phase for both the initial foam generation and then the prolonged gas injection. The surfactant solution was unsaturated, except in the case with a surfactant solution 60% saturated with CO_2_. The experiments have shown that the behavior of subsequently injected gas and liquid slugs in a SAG process was similar to that in this procedure [17].

The experiments on N_2_ foam were conducted in one Berea core, while the experiments on Kr foam and CO_2_ foam were performed in another one. The core was restored prior to starting the next experiment with a different gas as follows. First, 10 PVs of cleaning solution (a mixture of 50% isopropanol and 50% degassed water) was injected to destroy the foam inside the core. Then 20 PVs of degassed water was injected to displace the isopropanol in the core, followed by a period of CO_2_ injection to remove the remaining gas. Next, a vacuum was applied, followed by several short periods of CO_2_ injection, by imposing a vacuum on the core to remove any remaining gas, and by the injection of degassed water. The back pressure was subsequently elevated to 40 bar, and a volume of 10 PV of brine was injected to displace and dissolve the remaining CO_2_. The permeability of the core sample was measured after the core had been cleaned to ensure that it was in a comparable initial condition, with zero gas in the core.

## 4. Conclusions

In this work, we explored the impact of the gas composition on the injectivity of a surfactant solution in a surfactant-alternating-gas foam process. The findings are as follows.

- The strength of steady-steady foam was significantly influenced by the gas composition. In the high-quality regime, the apparent viscosities of N_2_ and Kr foam were close to each other and lower for CO_2_ foam.
- For all the gases tested, the flow pattern remained similar when the surfactant solution injection followed the foam injection. The initial surfactant solution injectivity was extremely poor. The pressure gradient plateau was heavily impacted by the initial strength of the foam. For stronger foam, the pressure gradient plateau during the surfactant solution injection was higher. The formation and evolution of liquid fingers, as well as the increase in surfactant solution injectivity, were primarily influenced by the solubility of gas in water. A higher gas solubility and a resulting greater gas dissolution capacity led to a faster decline in the pressure gradient (i.e., an increase in surfactant solution injectivity).
- The pattern of behavior observed during the gas injection in the SAG process was consistent for all the gases investigated in this study. The leading edge of the collapsed-foam zone advanced slowly from the entrance to the exit, resulting in a greater gas injectivity and a significant enhancement in subsequent surfactant solution injectivity. The velocity of the advancing front where the foam collapsed was dominated by the solubility of water in gas. The greater the water solubility in the gas, the swifter the advance of the collapsed-foam front.
- In a SAG process, surfactant solution injection involves the initial liquid filling of the collapsed-foam area produced by gas injection with a high level of mobility, and the subsequent penetration of the liquid through the weakened-foam area by fingering. This phenomenon occurred with all the gases studied here. Gas dissolution in the liquid was essential for the creation of liquid fingers and the increase in surfactant solution injectivity.

## Figures and Tables

**Table 1:** Gas solubility in water at 40 bar, 90 °C.

Gas	Solubility (mol/kg H_2_O)
N_2_	0.01
Kr	0.03
CO_2_	0.37

## Data Availability

The data that substantiate the conclusions of this study can be accessed openly through the following URL: https://doi.org/10.17632/66rg4fhfzf.1.
